# Quinetum and Its Sulphate

**Published:** 1877-01

**Authors:** 


Quinetum and its Sulphate.—Dr. J. Thorowgood desires to draw attention, says the *Medical Times and Gazette*, to the sulphate of quinetum as a useful tonic, suitable in some cases, it appears, where the sulphate of quinine does not agree. Quinetum itself, as prepared by Mr. T. Whiffin, of Battersea, is an amorphous pale brown powder, consisting of a mixture of the various alkaloids found in the East India red bark. From this quinetum the white crystallizable sulphate is prepared. The dose Dr. Thorowgood has lately been administering to his out-patients at the Victoria Park Hospital has ranged from one to three grains three times a day, held in solution with a few drops of dilute sulphuric acid. He has found the drug thus given to act in a very satisfactory way in promoting increase of appetite and strength in cases that had made at previous times but slow progress when treated with iron or bark. He had noted seven cases where the sulphate has been given for three weeks, and in every case it has agreed remarkably well as a general tonic, whilst in no case has any unpleasant result followed its employment. Sulphate of quinetum appears to embody all the valuable alkaloids of bark without any admixture of astringent or coloring matter. It is, moreover, cheaper than the sulphate of quinine.

